# Early onset of APC/C activity renders SAC inefficient in mouse embryos

**DOI:** 10.3389/fcell.2024.1355979

**Published:** 2024-03-13

**Authors:** Adela Horakova, Marketa Konecna, Lenka Radonova, Martin Anger

**Affiliations:** ^1^ Department of Genetics and Reproductive Biotechnologies, Veterinary Research Institute, Brno, Czechia; ^2^ Institute of Animal Physiology and Genetics, Academy of Sciences of the Czech Republic (ASCR), Libechov, Czechia; ^3^ Faculty of Science, Masaryk University, Brno, Czechia

**Keywords:** spindle, spindle assembly checkpoint, chromosome segregation, anaphase, embryo, Mad1, anaphase-promoting complex

## Abstract

Control mechanisms of spindle assembly and chromosome segregation are vital for preventing aneuploidy during cell division. The mammalian germ cells and embryos are prone to chromosome segregation errors, and the resulting aneuploidy is a major cause of termination of development or severe developmental disorders. Here we focused on early mouse embryos, and using combination of methods involving microinjection, immunodetection and confocal live cell imaging, we concentrated on the Spindle Assembly Checkpoint (SAC) and Anaphase Promoting Complex/Cyclosome (APC/C). These are two important mechanisms cooperating during mitosis to ensure accurate chromosome segregation, and assessed their activity during the first two mitoses after fertilization. Our results showed, that in zygotes and 2-cell embryos, the SAC core protein Mad1 shows very low levels on kinetochores in comparison to oocytes and its interaction with chromosomes is restricted to a short time interval after nuclear membrane disassembly (NEBD). Exposure of 2-cell embryos to low levels of spindle poison does not prevent anaphase, despite the spindle damage induced by the drug. Lastly, the APC/C is activated coincidentally with NEBD before the spindle assembly completion. This early onset of APC/C activity, together with precocious relocalization of Mad1 from chromosomes, prevents proper surveillance of spindle assembly by SAC. The results contribute to the understanding of the origin of aneuploidy in early embryos.

## Introduction

Mammalian oocytes and embryonic blastomeres are known to be prone to errors during chromosome segregation. Such errors might lead to chromosome loss or gain, and the resulting aneuploidy compromises the future development of the embryo or causes severe developmental disorders (Nagaoka et al., 2012). In oocytes, the origin of chromosome segregation errors was extensively studied in multiple species, including mice and humans. The problem, at least in mouse, seems to be caused by an inability to respond adequately to the spindle assembly defects, as reported by several laboratories, including our laboratory (Nagaoka et al., 2011; Gui and Homer, 2012; Kolano et al., 2012; Lane et al., 2012; Sebestova et al., 2012; Allais and FitzHarris, 2022). In human oocytes, chromosome segregation is even more compromised (Mihajlovic and FitzHarris, 2018; Charalambous et al., 2022). In early embryos, the mechanisms underlying chromosome segregation were not studied extensively, and thus, the reasons why embryonic blastomeres are frequently affected by aneuploidy are less clear (Vázquez-Diez and FitzHarris, 2018).

The embryos are usually mosaic, and the number of cells affected dictates the possibility of further development (Shahbazi et al., 2020). We have previously shown that in mouse 1–8-cell *in vivo* embryos, the frequency of aneuploidy per blastomere fluctuates between 4% and 7%, with a further increase of 11% during the 8–16-cell transition (Pauerova et al., 2020). Because of the increasing number of blastomeres within the embryo, such incidence of aneuploidy translates into more than half of the embryos carrying aneuploid blastomeres in the later stages of preimplantation development. Similar numbers were also reported in human (Carbone and Chavez, 2015), bovine (Destouni et al., 2016; Tšuiko et al., 2017), porcine (Hornak et al., 2012), and rhesus monkey embryos (Daughtry et al., 2019), indicating that aneuploidy represents a general problem for mammalian embryos.

Given the much higher frequency of aneuploidy in embryos compared to somatic cells, answering the question of whether chromosome segregation in early embryos fails more frequently or whether the control mechanisms are compromised is important. During mitosis, the spindle assembly and chromosome segregation are monitored through the spindle assembly checkpoint (SAC) pathway (Musacchio, 2015; Lara-Gonzalez et al., 2021; McAinsh et al., 2023). In response to the defects in the spindle apparatus and erroneous connections of kinetochores, SAC delays anaphase until all chromosomes are properly attached to the spindle. This ensures accurate segregation of chromosomes between daughter cells. SAC depends on the ability of unoccupied kinetochores to catalyze a conformational change in Mad2, which leads to the formation of the mitotic checkpoint complex (MCC). This complex, by sequestration of CDC20 from anaphase-promoting complex/cyclosome (APC/C), arrests cells in metaphase. Anaphase entry requires cessation of SAC activity, which is followed by swift APC/C activation. It is, however, not clear whether such a pathway also operates in early embryos and if so, whether it is fully functional.

In some metazoan species, such as ascidians, *Xenopus*, and zebrafish, SAC in embryos is active only after midblastula transition (MBT) (Clute and Masui, 1995; Zhang et al., 2015; Chenevert, 2020). In other species, such as *Caenorhabditis elegans*, SAC is already active before MBT (Encalada et al., 2005). In mammals, the situation is not very clear. Human embryos respond to nocodazole, and during later stages of development, the exposure leads to apoptosis (Jacobs et al., 2017). In mouse, the gene targeting studies showed that Mad2 and BubR1, which are both essential genes for the SAC pathway, did not prevent development to the blastocyst stage (Dobles et al., 2000). In addition, the deletion of various SAC proteins in zygotes affected chromosome segregation (Wei et al., 2011). Recently, SAC was also studied in mouse embryos, and the authors showed that 2-cell embryos exposed to nocodazole prolong mitosis and that during morula and blastocyst stages, the congression defects and lagging chromosomes are ignored (Vázquez-Diez et al., 2019).

Here, we assessed the SAC function during the first two mitotic divisions in mouse embryos. We focused on the ability of SAC to postpone APC/C activity. A combination of various techniques, such as immunodetection, live cell imaging, and pharmacological perturbations, revealed that SAC in early mouse embryos is unable to postpone anaphase in the case of defects in spindle assembly. This is consistent with the inability of embryos to effectively prevent chromosome segregation errors and aneuploidy.

## Results

### Detection of endogenous Mad1 on kinetochores in zygotes and 2-cell embryos

**FIGURE 1:**
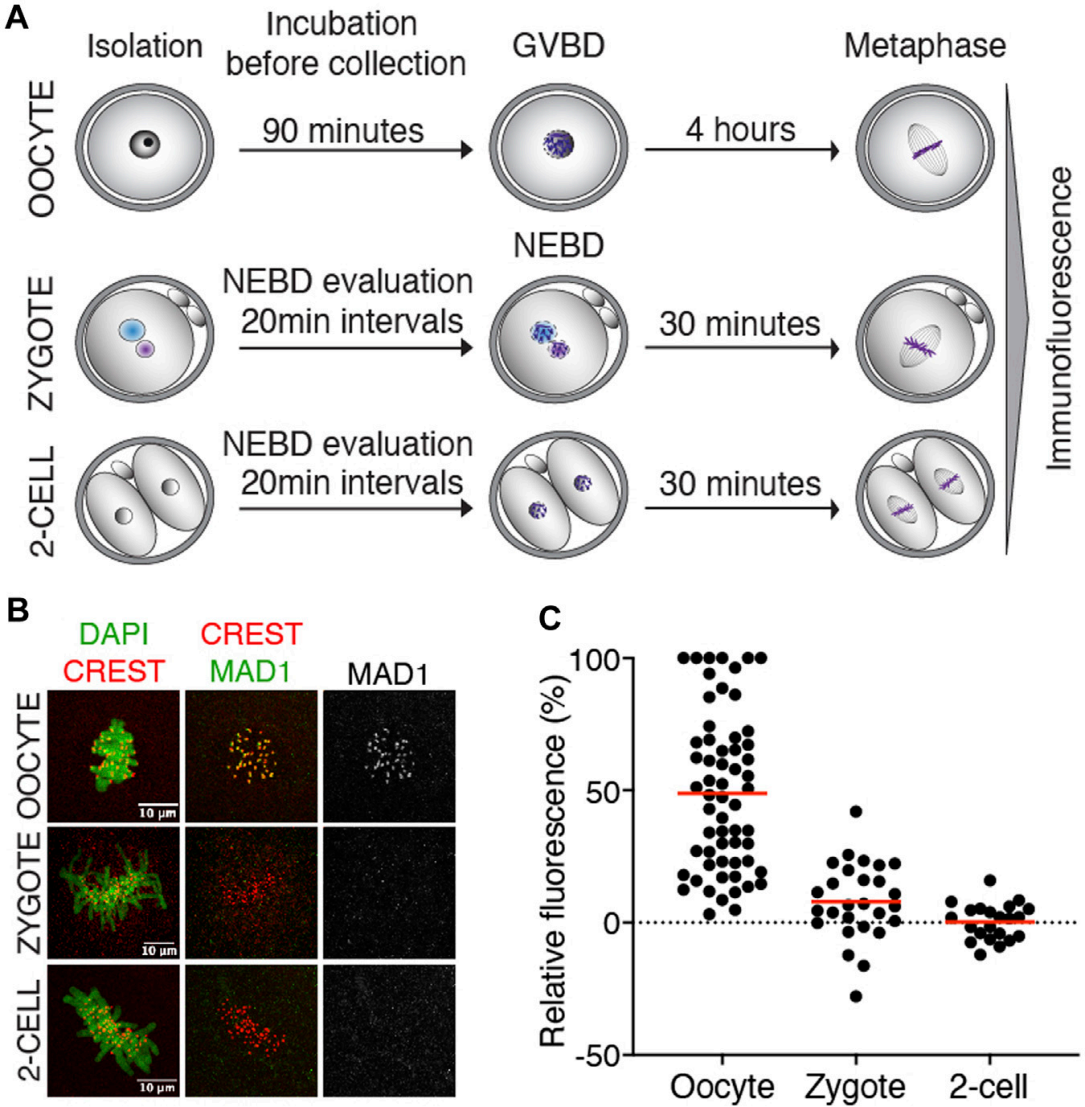
Endogenous Mad1 in oocytes and early embryos. **(A)** Scheme of synchronization protocol for obtaining oocytes and embryos in meiosis or mitosis. **(B)** Representative images of oocytes and early mouse embryos after immunostaining. DNA (green) was visualized by DAPI, centromeres were detected by the anti-CREST antibody (red), and Mad1 was detected by the anti-Mad1 antibody (green or gray). The scale bar represents 10 μm. **(C)** Scatterplot showing the signal of Mad1 detected by the antibody in control oocytes, zygotes, and 2-cell embryos. The average per group was 48.8 ± 29.5% in oocytes, 7.9 ± 14.5% in zygotes, and 0.3 ± 6.8% in 2-cell embryos. The difference between oocytes and zygotes or oocytes and 2-cell embryos was statistically significant (α < 0.05, ****p = <0.0001). The data were obtained from two independent experiments (oocytes: n = 60; zygotes: n = 27; 2-cell embryos: n = 22).

The function of SAC critically depends on the ability of Mad1/Mad2 to localize to unattached kinetochores, where it resides until connections to the spindle apparatus are established (Luo et al., 2018). Mad1 kinetochore localization, therefore, serves as a direct reporter of the local SAC activity. First, we focused on endogenous Mad1 to specifically determine whether the localization is consistent with its potential function in SAC. The Mad1 expression levels in zygotes and 2-cell embryos were compared to those in meiosis I oocytes with a known localization pattern (Zhang et al., 2005). For Mad1 detection, cells were collected in metaphase, meiosis I oocytes four hours after GVBD, and zygotes and 2-cell embryos 30–50 min after NEBD (Figure 1A). After processing for immunodetection of Mad1 and CREST, the signal of Mad1 was measured in the region of CREST localization (Supplementary Figure S1A), and the Mad1 cytoplasmic signal was subtracted. A comparison of the signal in zygotes and 2-cell embryos with oocytes showed significantly lower levels of endogenous Mad1 in embryos (Figures 1B, C).

### Inhibition of Mps1 has different consequences in zygotes and 2-cell embryos

**FIGURE 2:**
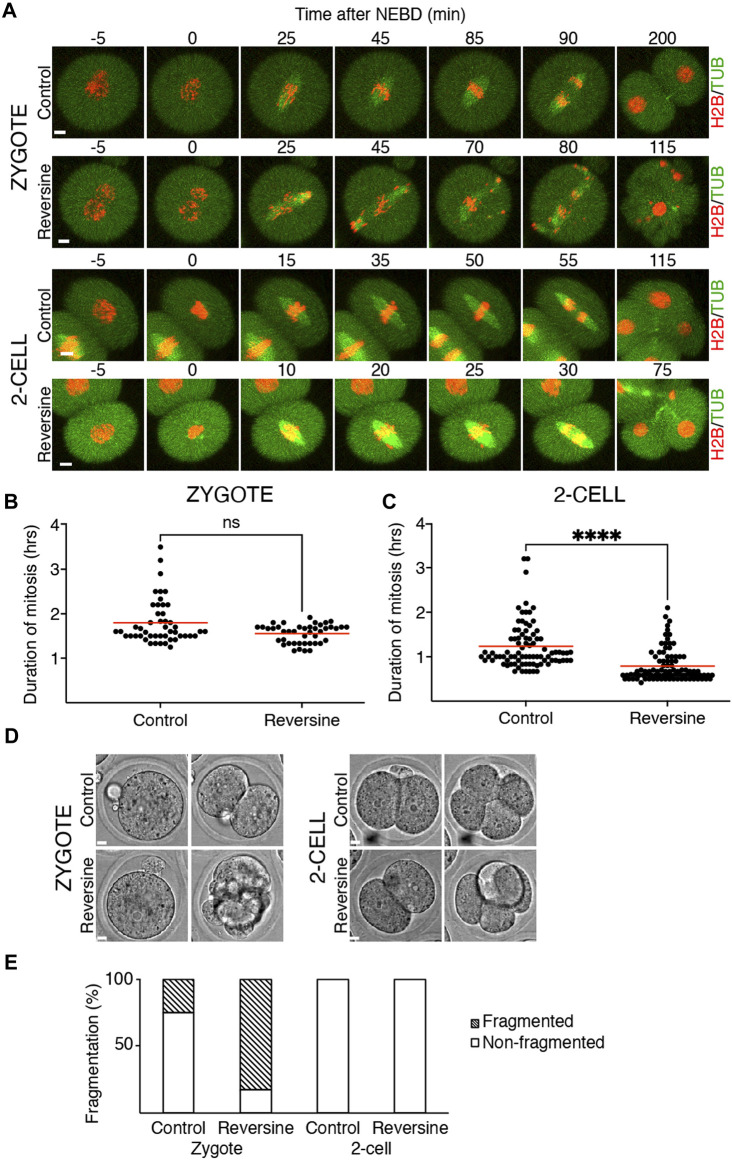
Reversine impacts chromosome segregation in oocytes and early mouse embryos. **(A)** Representative frames of mitosis from the time-lapse confocal microscopy of control and reversine-treated zygotes and 2-cell embryos. Cells were microinjected with histone H2B (red) and tubulin (green) fusion proteins. The scale bar represents 10 μm. **(B)** Scatterplot shows duration of mitosis from NEBD until anaphase in zygotes cultured without and with reversine (control: n = 48; reversine: n = 43). The average per group was 1.8 ± 0.5% in control zygotes and 1.55 ± 0.21% in reversine-treated zygotes. The difference between control and reversine-treated zygotes was not significant (*p* = <0.0991). The data were obtained from six experiments. **(C)** Scatterplot shows duration of mitosis from NEBD until anaphase in 2-cell embryos cultured without and with reversine (control: n = 86; reversine: n = 92). The average per group was 1.23 ± 0.52% in control 2-cell embryos and 0.79 ± 0.36% in reversine-treated 2-cell embryos. The difference between control and reversine-treated 2-cell embryos was significant (α < 0.05, ****p = <0.0001). The data were obtained from six experiments. **(D)** Representative images of division of control and reversine-treated zygotes and 2-cell embryos illustrating the fragmentation in zygotes. The scale bar represents 10 μm. **(E)** Fragmentation of control and reversine-treated zygotes and 2-cell embryos. The chart shows the percentage of fragmentation in the control group (zygotes: n = 24; 2-cell embryos: n = 46) and reversine group (zygotes: n = 23; 2-cell embryos: n = 47). In zygotes, the fragmentation was observed in 25% of the control group and 82.6% of the reversine group. The 2-cell embryos show no fragmentation. The chart shows data from three independent experiments.

Our previous results showed that the crucial SAC component Mad1 was almost absent in the kinetochores of zygotes and 2-cell embryos. Inhibition of SAC in mitosis (Shindo et al., 2021) or meiosis (McGuinness, 2009) causes M phase acceleration and increases the frequency of chromosome segregation defects. To inhibit SAC, we used reversine, a potent Mps1 kinase inhibitor (Santaguida et al., 2010), which was previously used in mouse oocytes (Hached et al., 2011; El Yakoubi et al., 2017) and embryos (Bolton et al., 2016). Mps1 inhibition should prevent Mad1 localizing to the kinetochores (Santaguida et al., 2010). Zygotes and 2-cell embryos were microinjected with cRNAs encoding tubulin-EGFP and histone H2B-mCherry fusion proteins. For all experiments, the identical concentration of reversine was employed for both embryos since they were in the same dish. The division was monitored by confocal microscopy. The results showed that reversine significantly affected chromosome segregation in both embryos, although it affected zygotes more severely (Figure 2A). In the majority of cells, the congression was profoundly affected, and chromosomes were scattered along the spindle axis. Whereas the length of mitosis in zygotes was unaffected by reversine, the length of mitosis in 2-cell embryos was accelerated by approximately one-third (Figures 2B, C). Mps1 inhibition accelerated meiosis I (Hached et al., 2011), reducing the timing to almost 50% of its normal duration. Such an acceleration is similar to the depletion of SAC components, for example, Bub1 (McGuinness, 2009) or BubR1 (Touati et al., 2015). Compared to oocytes, the effect of Mps1 inhibition on the duration of mitosis was apparent only in 2-cell embryos. Although the 2-cell embryos eventually divided, the division of zygotes was compromised and frequently resulted in cell fragmentation (Figures 2D, E). In conclusion, these experiments revealed different sensitivity of zygotes and 2-cell embryos to the pharmacological inhibition of SAC, with catastrophic consequences, but unchanged mitosis time length in zygotes and shortened mitosis in 2-cell embryos.

### Challenging SAC does not prevent anaphase in 2-cell embryos

**FIGURE 3:**
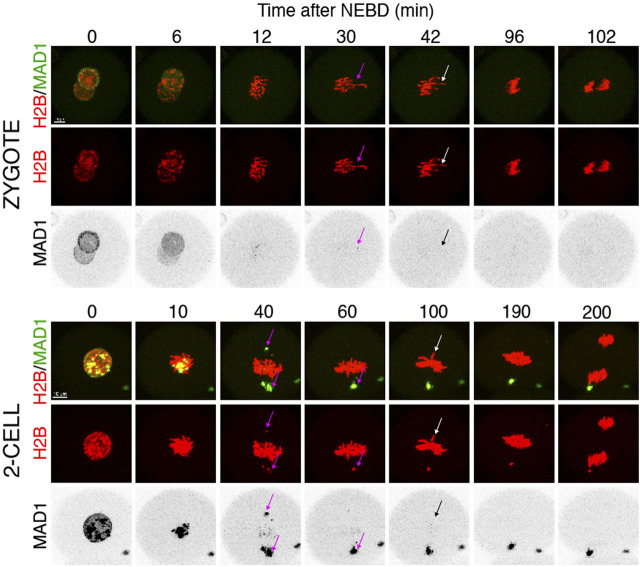
Mad1 signal on uncongressed chromosomes in zygotes and 2-cell embryos. Frames from the time-lapse experiments showing division of zygotes and 2-cell embryos from NEBD to anaphase. Early mouse embryos were microinjected with cRNAs encoding histone H2B (red) and Mad1 (green and black in the inverted part) fused to fluorescent proteins. The magenta arrow points to a chromosome with the Mad1 signal, whereas the white arrow (in inverted pictures black) points to a chromosome without the Mad1 signal. The scale bar represents 10 μm.

**FIGURE 4:**
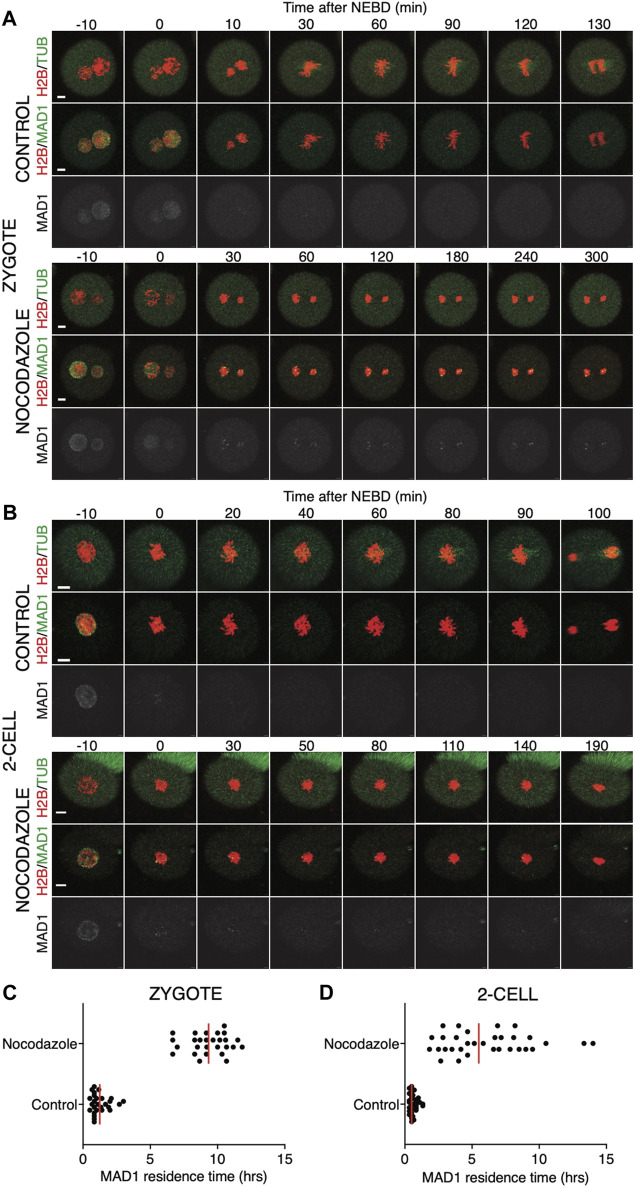
Impact of higher concentrations of nocodazole on division in zygotes and 2-cell embryos. **(A)** Representative mitosis of zygotes dividing with and without nocodazole. Embryos were microinjected with cRNAs encoding histone H2B (red), tubulin (green), and Mad1 (green and gray) fusion proteins. Cell division and chromosome segregation were assessed by time-lapse confocal microscopy. The scale bar represents 10 μm. **(B)** Representative mitosis of 2-cell embryos dividing with and without nocodazole. Embryos were microinjected with cRNAs encoding histone H2B (red), tubulin (green), and Mad1 (green and gray) fusion proteins. Cell division and chromosome segregation were assessed by time-lapse confocal microscopy. The scale bar represents 10 μm. **(C)** Scatterplot showing the residence time of the Mad1 signal on kinetochores throughout mitosis in zygotes. Time was scored in control zygotes (n = 27) and nocodazole-treated zygotes (n = 27). The presence of Mad1 was 1.25 ± 0.66% in control zygotes and 9.33 ± 1.47% in nocodazole-treated zygotes. The difference between groups was significant (α < 0.05, ****p = <0.0001). The data were obtained from three independent experiments. **(D)** Scatterplot showing the residence time of the Mad1 signal on kinetochores throughout mitosis in 2-cell embryos. Time was scored in control 2-cell embryos (n = 32) and nocodazole-treated 2-cell embryos (n = 24). The presence of Mad1 was 0.64 ± 0.3% in control embryos and 6.1 ± 3.1% in nocodazole embryos. The difference between groups was significant (α < 0.05, ****p = <0.0001). The data were obtained from three independent experiments.

Quantification of endogenous chromosomal Mad1 demonstrated its dramatically low levels on chromosomes in 1–2-cell embryos (Figure 1C). However, we could not exclude that a strong trigger, for example, uncongressed chromosomes, might cause Mad1 relocalization to the kinetochores. To address this, we assessed the Mad1 signal in zygotes and 2-cell embryos during a relatively rare event when chromosomes were visibly separated from the metaphase plate (Figure 3). The data were obtained from live cell imaging and showed, that Mad1 signal was not always associated with kinetochores of chromosomes outside of the metaphase plate. In another case, we show that the Mad1 signal was retained even during anaphase entry. Nevertheless, the overall frequency of congression defects in embryos was low and did not allow for drawing any conclusion. Therefore, we decided to challenge SAC by nocodazole (Figure 4). Cells were microinjected with tubulin-CFP, Mad1-Venus, and histone H2B-mCherry cRNAs and exposed to 266 nM nocodazole. Same as in reversine experiments, all cells were cultured in the same dish and therefore were exposed to the same concentration of the inhibitor. Confocal live cell imaging monitoring showed that all cells remained arrested in mitosis and had no spindle (Figures 4A, B). The Mad1 signal, albeit reduced, was detectable on chromosomes for a prolonged time interval (Figures 4C, D), which was on average longer in zygotes than in 2-cell embryos.

**FIGURE 5:**
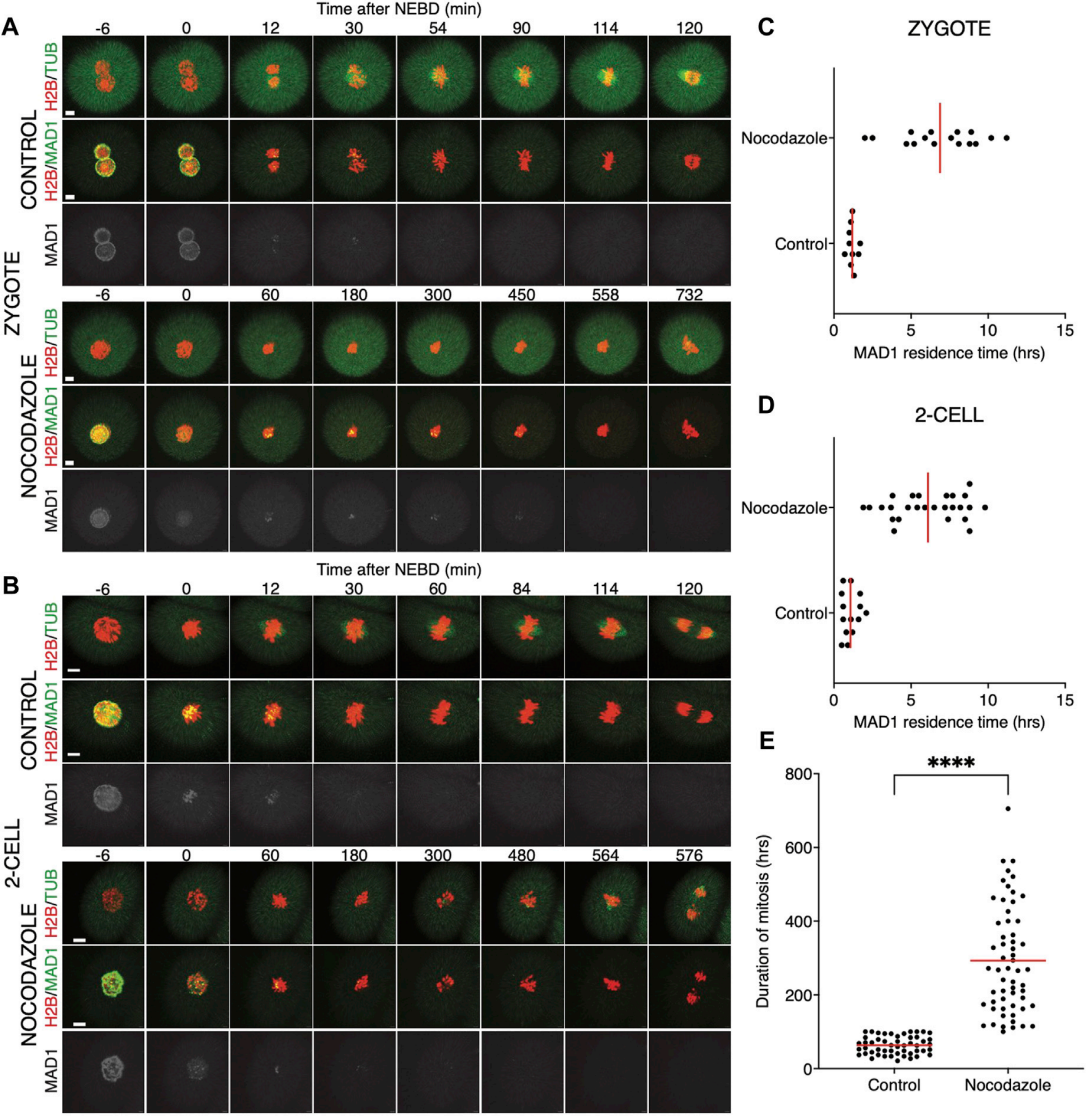
Low concentrations of nocodazole allows embryos to enter anaphase. **(A)** Representative mitosis of zygotes dividing with and without nocodazole. Embryos were microinjected with cRNAs encoding histone H2B (red), tubulin (green), and Mad1 (green and gray) fusion proteins. Cell division and chromosome segregation were assessed by time-lapse confocal microscopy. The scale bar represents 10 μm. **(B)** Representative mitosis of 2-cell embryos dividing with and without nocodazole. Embryos were microinjected with cRNAs encoding histone H2B (red), tubulin (green), and Mad1 (green and gray) fusion proteins. Cell division and chromosome segregation were assessed by time-lapse confocal microscopy. The scale bar represents 10 μm. **(C)** Scatterplot showing the residence time of the Mad1 signal on kinetochores throughout mitosis in zygotes. Time was scored in control zygotes (n = 10) and nocodazole-treated zygotes (n = 16). The presence of Mad1 was 1.19 ± 0.29% in control zygotes and 6.88 ± 2.60% in nocodazole-treated zygotes. The difference between groups was significant (α < 0.05, ****p = <0.0001). The data were obtained from two experiments. **(D)** Scatterplot showing the residence time of the Mad1 signal on kinetochores throughout mitosis in 2-cell embryos. Time was scored in control 2-cell embryos (n = 14) and nocodazole-treated 2-cell embryos (n = 26). The presence of Mad1 was 1.07 ± 0.52% in control embryos and 6.1 ± 2.28% in nocodazole embryos. The difference between groups was significant (α < 0.05, ****p = <0.0001). The data were obtained from two experiments. **(E)** Scatterplot of the duration of mitosis of 2-cell embryos with and without nocodazole (control 2-cell embryos: n = 51; nocodazole-treated 2-cell embryos: n = 58). The length of division in control 2-cell embryos was 63.2 ± 23.9%, and that in nocodazole-treated 2-cell embryos was 293.1 ± 145.5%. The difference between groups was significant (α < 0.05, ****p = <0.0001). The data were obtained from four experiments.

We also tested a lower 100 nM concentration of nocodazole (Figure 5) in the same experimental setup. A lower concentration of the drug will allow for building the spindle, but it should challenge the SAC. The spindle morphology was, however, significantly affected by nocodazole, more so in zygotes than in 2-cell embryos (Figures 5A, B). The spindle assembly in zygotes therefore seems to be more sensitive to tubulin depolymerization than that in 2-cell embryos. The residence time of Mad1 on kinetochores was extended (Figures 5C, D) and was comparable to the higher doses of the drug (Figures 4C, D). The main difference therefore was in the presence of spindle apparatus in cells exposed to a lower dose. Although the 2-cell embryos eventually divided, they spent a much longer time in division compared to the control embryos (Figure 5E). Importantly, this experiment revealed that the 2-cell embryos were able to execute chromosome division even with visibly damaged spindles and that Mad1 was displaced from the chromosomes only just before anaphase entry.

### Early onset of APC/C activity in 2-cell embryos after entry into mitosis

**FIGURE 6:**
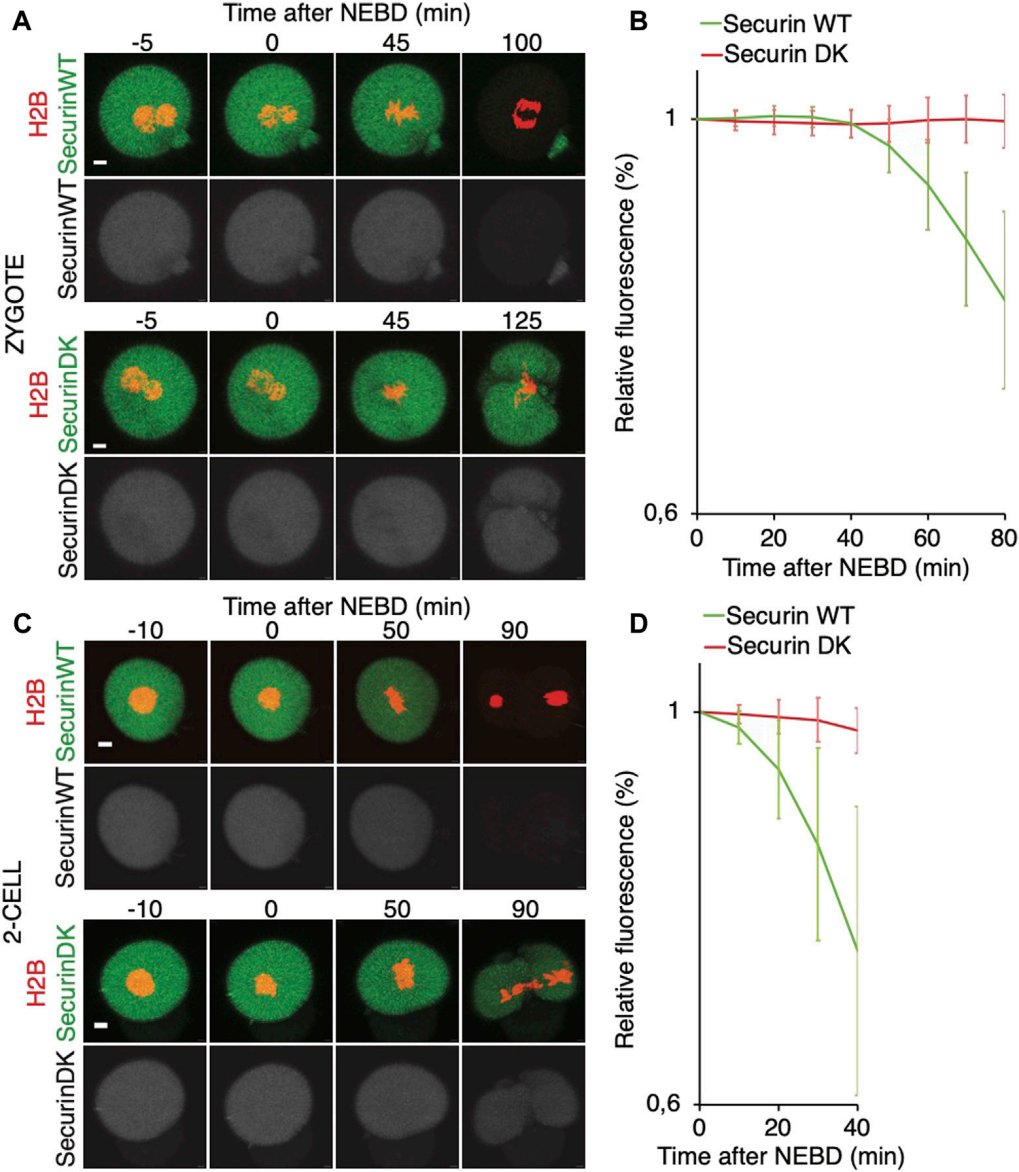
Onset of securin destruction in zygotes and 2-cell embryos. **(A)** Zygotes were microinjected with cRNAs encoding WT securin or securin with mutated K/D boxes (securin DK) and histone H2B (red) fused to fluorescent protein, and subsequently, the fluorescence signal was measured by time-lapse confocal microscopy. The scale bar represents 10 μm. **(B)** Fluorescence signal profiles of securin WT (green, n = 56) and securin DK (red, n = 52) during mitosis I. The graph shows the average relative values from NEBD to the shortest anaphase. The data were obtained from four independent experiments. **(C)** 2-cell embryos were microinjected with cRNAs encoding WT securin or securin with mutated K/D boxes (securin DK) and histone H2B (red) fused to fluorescent protein, and subsequently, the fluorescence signal was measured by time-lapse confocal microscopy. The scale bar represents 10 μm. **(D)** Fluorescence signal profiles of securin WT (green; n = 11) and securin DK (red; n = 11) in 2-cell embryos. The graph shows the average relative values from NEBD to the shortest anaphase. The data were obtained from two independent experiments.

**FIGURE 7:**
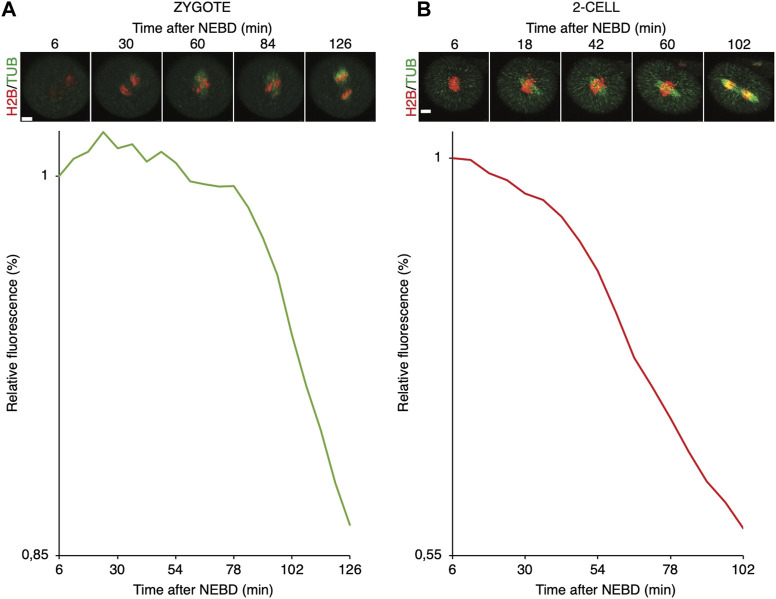
Relationship between the spindle assembly and APC/C activity in mouse zygotes and 2-cell embryos. **(A)** Upper panel represents selected frames from the time-lapse experiment showing the division of representative zygote. Cell was microinjected with cRNAs encoding histone H2B (red), tubulin (green), and 90 cyB1 (not shown) fused to fluorescent proteins. The scale bar represents 10 μm. The lower panel shows fluorescence levels of 90 cyB1 during mitosis of the same cell. **(B)** Upper panel represents selected frames from the time-lapse experiment showing division of representative 2-cell blastomere. The cell was microinjected with cRNAs encoding histone H2B (red), tubulin (green), and 90 cyB1 (not shown) fused to fluorescent proteins. The scale bar represents 10 μm. The lower panel shows fluorescence levels of 90 cyB1 during mitosis of the same cell.

It was shown that in zygotes, the destruction of cyclin B is delayed, whereas in 2-cell embryos, the cyclin B destruction seems to be initiated earlier (Ajduk et al., 2017). Here, we used securin, which is also an APC/C substrate, to assess a link between spindle assembly and APC/C activity (Figure 6). For our experiments, we used tagged wild-type securin (WT securin), as well as tagged securin with mutations in KEN box and D box (DK securin), which should render securin resistant to APC/C (Thomas et al., 2021). Our results showed that the destruction of securin in 2-cell embryos starts immediately after NEBD, whereas in zygotes, it is delayed (Figures 6A–D). The DK securin showed that the onset of securin destruction in zygotes and 2-cell embryos is dependent on APC/C. Importantly, although in zygotes, the onset of securin destruction was delayed until the completion of the spindle assembly and establishment of the metaphase plate, the link between the initiation of securin destruction and completion of spindle assembly in 2-cell embryos is lost (Figures 7A, B).

**FIGURE 8:**
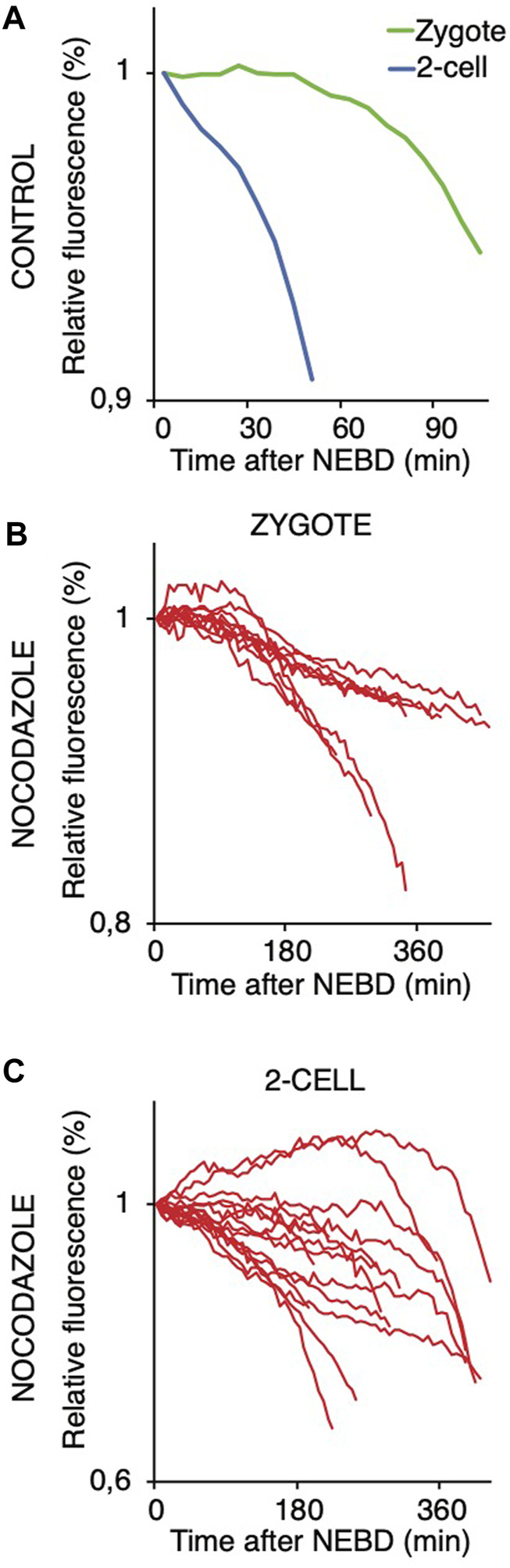
Targeting 1–90 cyB1 for destruction after NEBD in zygotes and 2-cell embryos with and without nocodazole. **(A)** Fluorescence signal profiles of 90 cyB1 in zygotes (green; n = 13) and 2-cell embryos (blue; n = 20) during mitosis. The graph shows the average relative values from NEBD to the shortest anaphase. The data were obtained from two independent experiments. **(B)** Fluorescence signal profiles of 90 cyB1 in zygotes (red; n = 10) during mitosis I. The graph shows the relative values from NEBD to the anaphase. The data were obtained from two independent experiments. **(C)** Fluorescence signal profiles of 90 cyB1 in 2-cell embryos (red; n = 15) during mitosis II. The graph shows the relative values from NEBD to the anaphase. The data were obtained from two independent experiments.

We also compared the stability of cyclin B in zygotes and 2-cell embryos (Figure 8). For this, we used the first 90 amino acids of cyclin B1 (1–90 cyB1) that contain the D box but lack the ability to interact with CDK1 (Glotzer et al., 1991), which minimizes the risk of mitosis perturbation. Our results showed that similar to the full-length cyclin, the destruction of 1–90 cyB1 fragments was delayed in zygotes but commenced immediately after NEBD in 2-cell embryos (Figure 8A). We also tested the ability of nocodazole to prevent APC/C activation and destruction of 1–90 cyB1. Our results showed that in zygotes and 2-cell embryos exposed to 100 nM nocodazole (lower dose, allowing the 2-cell embryos to divide), the destruction of 1–90 cyB1 was slower in the majority of cells but not abolished (Figures 8B, C). A prolonged residence time of Mad1 was shown at this concentration of nocodazole (Figures 5C, D). Therefore, the APC/C seems to be active in these cells despite the association between Mad1 and chromosomes.

## Discussion

In dividing cells, the control of spindle assembly, more specifically the control of microtubule–kinetochore connections, is of paramount importance for the fidelity of chromosome segregation (Curtis et al., 2020; Lara-Gonzalez et al., 2021; McAinsh et al., 2023). The importance of these surveillance mechanisms is undisputable as potential errors in chromosome segregation have severe consequences in daughter cells and in the case of multicellular organisms, for the entire body. However, mammalian germ cells and embryos suffer from exceptionally high incidences of chromosome segregation errors. In addition, the aneuploidy in these cells leads to the termination of development or causes severe developmental disorders (Hassold and Hunt, 2001; Nagaoka et al., 2012; Mihajlovic and FitzHarris, 2018; Charalambous et al., 2022).

The canonical function of SAC is to postpone APC/C activation until the completion of spindle assembly when all kinetochores are correctly attached to the spindle microtubules and the sister kinetochores are oriented to the opposite spindle poles (Musacchio, 2011). Once this is achieved, the SAC signaling turns off, which leads to the activation of APC/C and ubiquitination and proteolysis of cyclin B, securin, and other substrates. Therefore, during entry into mitosis, the CDC20-controlled substrates should be, at least initially, stabilized until the completion of spindle assembly and degraded afterward. This important ability of SAC to postpone APC/C activation is a cornerstone of chromosome segregation control, and its function was demonstrated in HeLa cells (Clute and Pines, 1999) and confirmed in many cell types thereafter.

Our data presented here show that during unperturbed division, mouse zygotes fully activate APC/C after the spindle assembly is completed, whereas the 2-cell embryos fully activate APC/C much earlier, simultaneously with NEBD. Our data in this regard are similar to a previous report (Ajduk et al., 2017). Here, we used 2 different substrates, securin and a fragment of cyclin B, to confirm this, with both showing similar results. As a consequence, the spindle is assembled when the APC/C is already fully active, and this might be the reason why the embryos lose the ability to respond to single uncongressed chromosomes (our results here and also Vázquez-Diez et al. (2019)). It does not, however, exclude a possibility of SAC reactivation by a strong impulse, such as spindle damage, induced after the initiation of spindle assembly. This might reactivate SAC even during ongoing APC/C activity. Nevertheless, it needs to be tested since in our experiments, we applied nocodazole before mitotic entry.

Using lower concentrations of nocodazole, we show that the SAC is unable to arrest cells in metaphase in response to substantial spindle damage. Although with significant delay, the cells were able to enter into anaphase. In this situation, it is difficult to assess whether the delay was caused by prolonged SAC activity. However, the gradual loss of the Mad1 signal from chromosomes during prolonged mitosis would perhaps favor the alternative explanation that the delay might rather be caused by the direct effect of nocodazole on spindle microtubules. During the time of nocodazole-induced mitotic arrest, in the majority of cells, the fragment of cyclin B is not completely stabilized but instead slowly degraded. This would also support our view that even strong activation of SAC, which is sufficient to keep the fraction of Mad1 on chromosomes, is not capable of preventing the destruction of the cyclin B fragment. On the other hand, the mutations in securin APC/C-dependent sequences prevented its degradation completely. The slow degradation of the cyclin B fragment is similar to its degradation during the process, which allows cells to escape from SAC arrest, which is called mitotic slippage (Lok et al., 2020). In that case, it would be another argument for weak SAC in embryos because the duration of arrest induced by lower nocodazole levels was up to 10 h, which is usually shorter compared to the 24-h arrest in somatic cells.

The conclusion drawn from APC/C activation is supported by Mad1 expression and localization in embryos. This protein was found to be essential for SAC functionality in all cells studied so far (Luo et al., 2018). Despite its indispensability for SAC, it shows low expression levels in both zygotes and 2-cell embryos. Its interaction with chromosomes takes place during unperturbed divisions restricted to a very narrow time interval, following NEBD. In addition, although we show that the exposure to nocodazole prolongs Mad1 interaction with chromosomes, when the levels of the nocodazole are low, allowing the assembly of residual spindles, the Mad1 loses its localization to chromosomes after prolonged mitosis anyway, and the cells initiate anaphase. We believe that this is another strong evidence that the SAC in embryos is unable to prevent anaphase for a long time, even in the situation when the spindle is substantially damaged. We still could not exclude that low expression of Mad1 in 2-cell embryos and perhaps other molecules from the SAC pathway is simply insufficient to produce enough MCC to delay APC/C activation. However, our data with overexpressed Mad1 indicate that rather than the expression itself, the association with kinetochores could be the problem.

Pharmacological inhibition of Mps1 is an effective way to prevent SAC activity. In our experiments, we used reversine, a potent Mps1 kinase inhibitor (Santaguida et al., 2010). Previously published results using different inhibitors showed that in zygotes, Mps1 inhibition negatively impacts chromosome segregation and compromises further development (Ju et al., 2021). In our experiments, we observed a much stronger effect. Chromosome segregation in zygotes was severely affected, and cells fragmented at anaphase. In contrast to the zygotes, 2-cell embryos were able to execute anaphase in the same concentration of the inhibitor. On the other hand, the length of their mitoses was significantly shorter. We can speculate to what extent the effect we observed in zygotes might have been caused by the side effects of reversine. This small molecule was originally discovered as an inhibitor of aurora kinases, and it was later found to inhibit Mps1 more potently than auroras (Hiruma et al., 2016). The assembly of the spindle in zygotes is, however, a more complex process than in ordinary cells because it involves a stage during which paternal genomes build their spindles which later join into a single spindle (Reichmann et al., 2018). It is conceivable that partial inhibition of aurora kinases, which are crucial for spindle assembly and function, might prevent the formation of the definitive spindle prior to division and be responsible for the phenotype we observed in zygotes.

We believe that our results support the overall conclusion that mouse 2-cell embryos normally do not require SAC activity for progression during mitosis. This is similar to some other vertebrates, in which the SAC is activated only after MBT, such as *Xenopus* and zebrafish (Clute and Masui, 1995; Zhang et al., 2015). In contrast to those species, however, we show that in mice, exposure to nocodazole delays anaphase and slows down the degradation of cyclin B. It remains to be tested, however, whether the activation of SAC within mitosis would be sufficient to revert APC/C activity.

## Materials and methods

### Animals, isolation, and culture of oocytes and embryos and inhibitors

CD-1 mice were purchased from the Animal Breeding and Experimental Facility, Faculty of Medicine, Masaryk University, Brno, Czech Republic; BDF1 males were purchased from AnLab, Prague, Czech Republic. CD-1/BDF1 mice were obtained by crossing CD-1 female mice and BDF1 male mice. For all experiments, at least 3-month-old females were used. All animal work was conducted according to Act No 246/1992 Coll. on the protection of animals against cruelty and was approved by the Central Commission for Animal Welfare, under approval ID 37/2021. The methods for the collection of oocytes and embryos were described previously (Pauerova et al., 2021). In brief, to collect embryos, mice were stimulated with 5 IU of pregnant mare serum gonadotropin (PMSG; BioVendor, Brno, Czech Republic), followed by stimulation with 5 IU of human chorionic gonadotropin (hCG; Merck, Darmstadt, Germany) 44–48 h later, and mated with BDF1 males. Zygotes and 2-cell embryos were isolated 18–27 and 41–46 h after hCG stimulation by the manual rupture of the oviduct in M2 medium, respectively, and zygotes were incubated with 1 mg/mL hyaluronidase (Merck) in M2 medium for cumulus cell removal. We cultured embryos in KSOM (Merck) covered with mineral oil (Nidacon, Mölndal, Sweden) at 37 °C and 5% CO_2_. GV-stage oocytes were isolated in M2 (Merck) and cultured in M16 media (Merck). In some experiments, oocytes and embryos were treated with 500 nM reversine (Merck) or with 100 nM and 266 nM of nocodazole (Merck) diluted in KSOM (Merck).

### Microinjection of GV oocytes and embryos

The microinjection of mouse oocytes and embryos was performed in M2 media (Merck), as previously described (Pauerova et al., 2021). The cRNAs used were as follows: securin-CFP, securin-EGFP, securin-DK EGFP, a fragment of the first 90 aa of cyclin B1 (1–90 cyB1) fused to Venus, tubulin-CFP, tubulin-EGFP, Mad1-Venus, histone H2B-mCherry, and histone H2B-mPlum. cRNAs for microinjection were diluted in RNase-free water to a concentration of 2–4 ng per μL.

### Immunodetection

The immunofluorescence protocol was described by Pauerova et al. (2021). In brief, oocytes and embryos were harvested after the previous culture. The zona pellucida was removed with acid Tyrode solution (Merck). Cells were fixed in 2% paraformaldehyde (Merck) for 20 min, permeabilized with 0.1% Triton X-100 (Merck) for 15 min, and blocked for 15 min at RT. The antibodies rabbit anti-Mad1 (1:200 dilution, GeneTex, Irvine, CA, United States), human anti-CREST (1:500 dilution, ImmunoVision, Springdale, AR, United States), Alexa Fluor 488 goat anti-rabbit (1:500 dilution, Thermo Fisher Scientific, Waltham, MA, United States), and Alexa Fluor 647 goat anti-human (1:500 dilution, Thermo Fisher Scientific) were used. VECTASHIELD with DAPI (Vector Laboratories, Burlingame, CA, United States) was used for mounting on microscope slides.

### Microscopy and live cell imaging

Fixed sample staining was scanned on the Olympus FV3000 confocal microscope equipped with an HCPL APO 60XS2/1.30 silicone immersion objective and HSDs. Absorbance at 405 nm, 488 nm, and 640 nm excitation wavelengths was measured using HSDs for the detection of DAPI, Alexa Fluor 488, and Alexa Fluor 647, respectively. Imaging of live cells was performed on a Leica SP5 confocal microscope and Olympus FLUOVIEW 3000 confocal microscope, both equipped with an EMBL incubator (37°C; 5% CO_2_) (EMBL, Heidelberg, Germany). The following objectives were used: Leica HCX PL APO ×40/1.1 water immersion objective and Olympus UPLSAPO 30xs/1.05 silicone immersion objective. Absorbance at 458 nm or 445 nm, 488 nm, 514 nm, and 561 nm excitation wavelengths were measured using hybrid detectors (Leica) and HSDs (Olympus) for the detection of CFP, EGFP, Venus, and mCherry fluorescent proteins.

### Image analysis and statistical analysis

Fiji (ImageJ, National Institutes of Health, Bethesda, MA, USA), LAS AF (http://www.leica-microsystems.com), and Imaris software (www.bitplane.com) was used for image analysis. For the quantification of the securin and 1–90 cyclin B1 signal the expression level was normalized to the level at GVBD or NEBD, as previously (Pauerova et al., 2021). To quantify the Mad1 signal on the kinetochores, the CREST signal was used to define the ROI. The background of the area with the same size was subtracted from the level of the Mad1 fluorescence signal on the kinetochore area. GraphPad Prism was used for the statistical testing of data. To analyze the difference between groups, the Mann–Whitney test was used.

## Data Availability

The original contributions presented in the study are included in the article/Supplementary Material; further inquiries can be directed to the corresponding author.
