# Supplementary figures and images for: Early onset of APC/C activity renders SAC inefficient in mouse embryos
Source: Front Cell Dev Biol. 2024 Mar 13;12:1355979. doi: 10.3389/fcell.2024.1355979 (PMC10965688; doi:10.3389/fcell.2024.1355979)

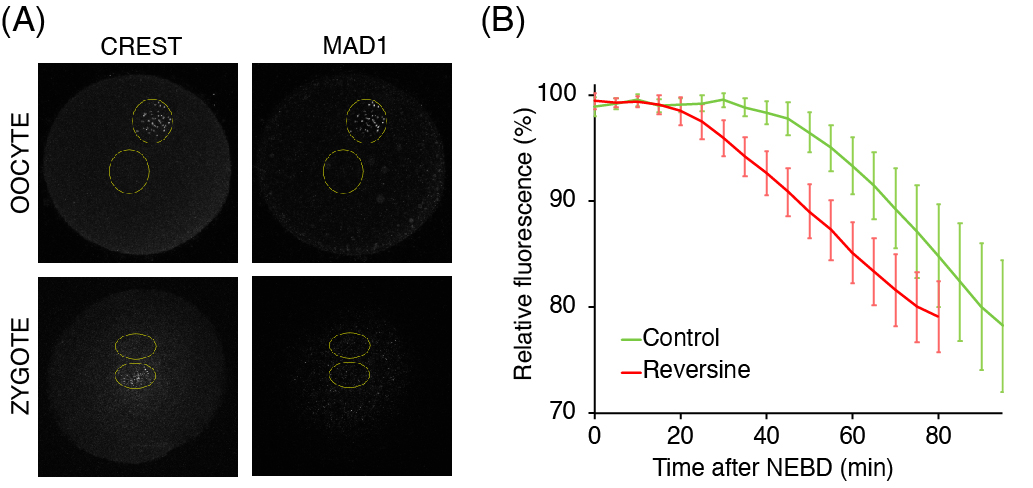

Supplement: Supplementary file 1 [file Image1.jpg]
